# Identification of radiologic and clinicopathologic variables associated with tumor regression pattern and distribution of cancer cells after short-course radiotherapy and consolidation chemotherapy in patients with rectal cancer

**DOI:** 10.3389/fonc.2024.1386697

**Published:** 2024-06-21

**Authors:** Alexandre Gheller, Dunya Bachour Basílio, Marília Cristina Rosa da Costa, Sussen Araújo Tuma, Oscar Miguel Túlio Andrade Ferreira, Fernando Gonçalves Lyrio, Daniel da Motta Girardi, João Batista de Sousa

**Affiliations:** ^1^ Colorectal Surgery Department, Hospital de Base do Distrito Federal, Brasília, DF, Brazil; ^2^ Anatomopathology Department, Hospital de Base do Distrito Federal, Brasília, DF, Brazil; ^3^ Clinical Oncology Department, Hospital de Base do Distrito Federal, Brasília, DF, Brazil; ^4^ Division of Colorectal Surgery, Universidade de Brasília (UnB), Brasília, DF, Brazil

**Keywords:** rectal cancer, fragmentation, distribution, tumor response, neoadjuvant (chemo)radiotherapy

## Abstract

**Background:**

Knowledge of the pattern of regression and distribution of residual tumor cells may assist in the selection of candidates for rectum-sparing strategies.

**Objective:**

To investigate and identify factors associated with tumor regression pattern and distribution of residual tumor cells.

**Methods:**

We conducted a prospective study of patients with T3/T4 N0/N+ adenocarcinoma of the middle and lower third of the rectum (≤10 cm) treated with radiotherapy (5×5 Gy) followed by 6 cycles of CAPOX chemotherapy. The pattern of tumor regression was classified as fragmented or solid. Microscopic intramural spread was measured. We used a model of distribution of residual tumor cells not yet applied to rectal cancer, defined as follows: type I (luminal), type II (invasive front), type III (concentric), and type IV (random).

**Results:**

Forty patients were included with a median age of 66 years; 23 (57.5%) were men. A fragmented pattern was identified in 18 patients (45.0%), and a solid pattern in 22 (55.0%). Microscopic intramural spread was identified in 25 patients (62.5%), extending from 1 to 18 mm (median, 4 mm). There were 14 cases (35.0%) of microscopic intramural spread ≥10 mm. All cases of fragmented regression pattern, except one, showed microscopic intramural spread. Within the fragmented pattern, microscopic intramural spread was 4–8 mm in 4 cases and ≥10 mm in the remaining cases. All cases of microscopic intramural spread ≥ 10 mm were within the fragmented pattern. Regarding the distribution pattern of residual tumor cells, 11 cases (31.5%) were classified as type I, 14 (40.0%) as type II, 10 (28.5%) as type III, and none as type IV. Carcinoembryonic antigen levels >5 ng/mL, downsizing <50%, residual mucosal abnormality >20 mm, and anatomopathologic lymph node involvement were significantly associated with the occurrence of fragmentation (*P*<0.05). Having received all 6 cycles of CAPOX chemotherapy and absence of microscopic intramural spread were significantly associated with the type I distribution pattern (*P*<0.05).

**Conclusion:**

The occurrence of a fragmented regression pattern is common, as is the presence of microscopic intramural spread. We could identify radiologic and clinicopathologic factors associated with the pattern of tumor regression and a type I distribution pattern.

## 1 Introduction

The treatment of rectal cancer has undergone a substantial change in recent decades. The surgical technique, total mesorectal excision (TME), has been standardized and associated with decreased local recurrence and increased overall survival (1, 2). The subsequent use of preoperative (neoadjuvant) chemoradiotherapy has further reduced the rate of local recurrence permitting more sphincter-sparing operations to be performed (3).

Neoadjuvant chemoradiotherapy leads to a complete destruction of tumor cells in up to one-third of cases (pathologic complete response [pCR]) (4, 5), which has allowed the implementation of rectum-sparing strategies, such as local excision and watch and wait (6, 7). In this scenario, proper identification of candidates can be challenging.

To apply rectum-sparing strategies, homogeneous tumor regression is advisable, where tumor cells are destroyed from deeper layers toward the lumen, with a consequent correspondence between gross and microscopic tumor. Unfortunately, this is not what happens in all cases. Up to 50% of tumors show a fragmented regression pattern, with clusters of tumor cells spreading across the different layers of the rectal wall (8–10).

The identification of a pattern of distribution and regression may facilitate the selection of potential candidates for organ-sparing approaches, as well as for radical surgery. However, few articles have described how residual tumor cells (RTCs) are distributed throughout the rectum and how the tumor regresses (11, 12).

Therefore, this study aimed to investigate the pattern of regression and distribution of RTCs and identify possible associated factors.

## 2 Methods

The study protocol was approved by the Research Ethics Committee of the Institute of Strategic Health Care Management of the Federal District – Hospital de Base of the Federal District, Brazil. No changes to the study protocol or methods were made after study commencement.

We conducted a prospective study of consecutive patients with extraperitoneal T3/T4 N0/N+ rectal adenocarcinomas, located 10 cm or less from the anal verge, who were treated and operated on at the Hospital de Base of the Federal District from January 2022 to June 2023. Patients were excluded if they had synchronous colorectal cancers or other non-colorectal cancers, had stage IV disease, had rectal cancer in the setting of inflammatory bowel disease or familial adenomatous polyposis, had undergone palliative resection, had previously received radiotherapy or chemotherapy, showed loss of expression of DNA repair enzymes, or had achieved a clinical complete response (cCR).

For all patients, baseline assessment and staging included a thorough proctoscopic examination, colonoscopy, dedicated pelvic magnetic resonance imaging (MRI) for rectal cancer, computed tomography of the chest and abdomen, and measurement of carcinoembryonic antigen (CEA) levels. Restaging was performed in all patients at the end of chemotherapy, between 1 and 6 weeks before surgery, and consisted of a thorough proctoscopic examination, flexible rectosigmoidoscopy, and pelvic MRI. During staging and restaging, information provided by pelvic MRI scanning was standardized according to the National Comprehensive Cancer Network (NCCN) Guidelines for Rectal Cancer (13). On restaging MRI, information was added on downsizing ≥ 50% (defined as an at least 50% reduction in the largest tumor diameter) and downstaging (stage regression).

Neoadjuvant therapy consisted of short-course radiotherapy (5 × 5 Gy) followed by 6 cycles of CAPOX chemotherapy (capecitabine 1000 mg/m^2^ orally twice daily on days 1–14, oxaliplatin 130 mg/m^2^ intravenously on day 1, and a chemotherapy-free interval between days 15–21) (14). Patients who achieved a cCR (absence of identifiable tumor on digital rectal examination, endoscopic ultrasound, and MRI) were offered the watch-and-wait strategy. Patients underwent surgery between 2 and 8 weeks after the end of chemotherapy. Surgery consisted of TME with or without sphincter preservation.

### 2.1 Anatomopathologic examination

**Figure 1:**
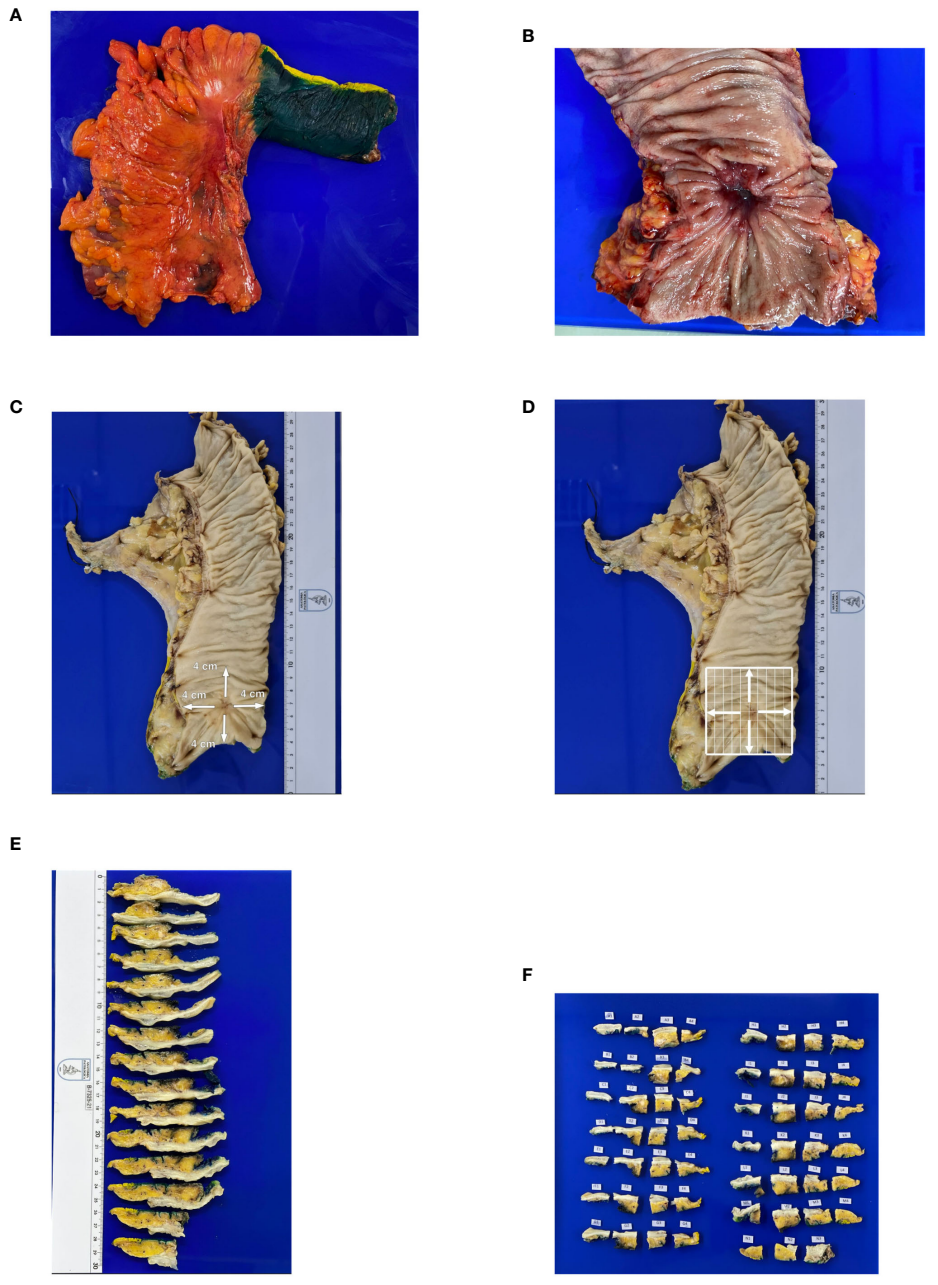
Standardization of gross examination of surgical specimens. Specimen **(A)** inking and **(B)** opening. **(C, D)** Specimen marked in 4 directions from the center of the residual tumor for a 4 × 4 cm square field. **(E)** Cross sections and **(F)** mapping.

**Figure 2:**
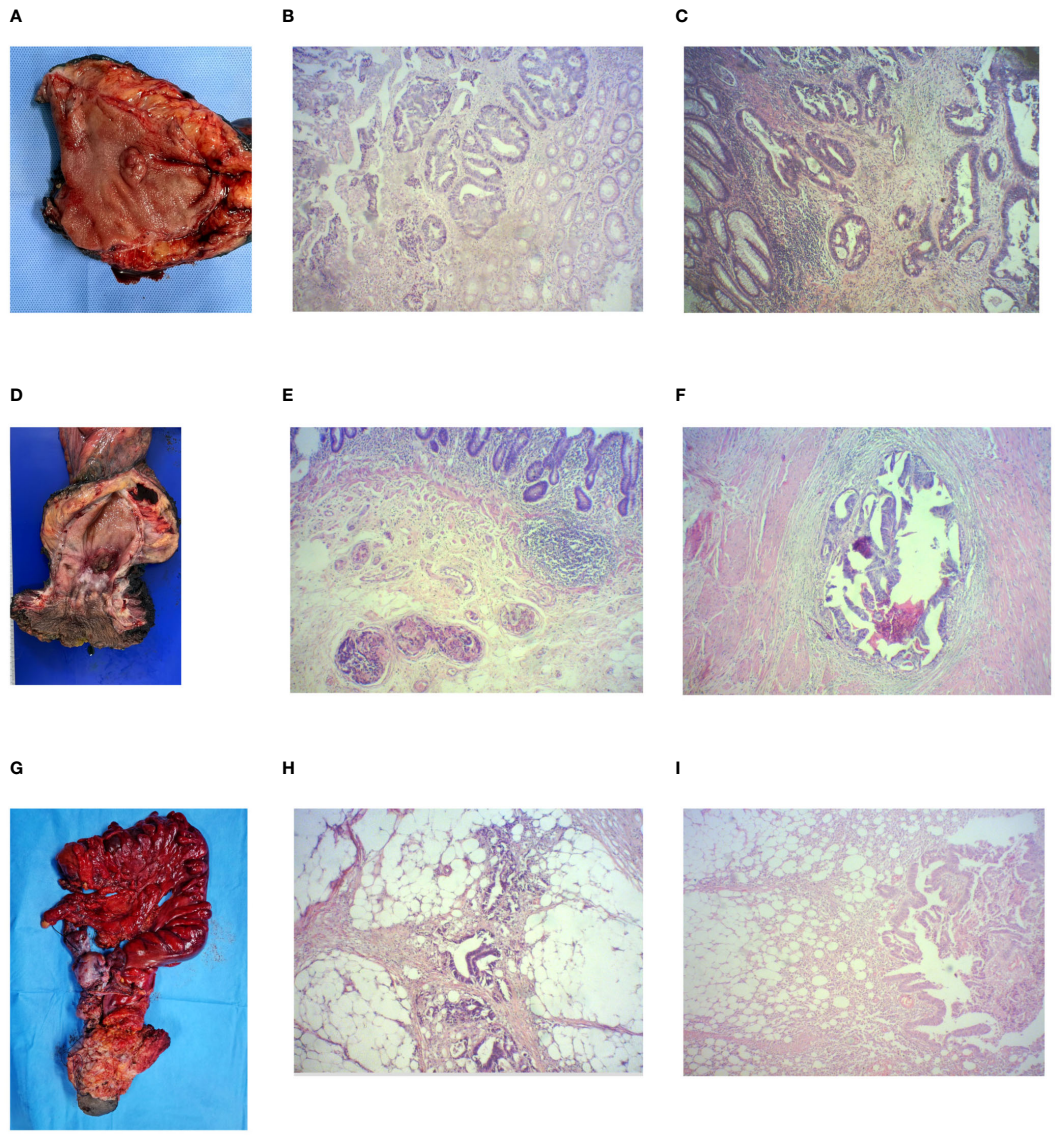
Surgical specimens with their pattern of tumor regression and distribution of residual tumor cells. **(A)** Resected specimen of low anterior open rectal resection: **(B)** solid regression pattern and **(C)** type I distribution pattern (hematoxylin & eosin, 40× magnification). **(D)** Resected specimen of open abdominoperineal resection: **(E)** fragmented regression pattern and **(F)** type III distribution pattern (hematoxylin & eosin, 40× magnification). **(G)** Resected specimen of infralevator posterior pelvic exenteration with combined sacrectomy: **(H)** fragmented regression pattern and **(I)** type II distribution pattern (hematoxylin & eosin, 40× magnification).

A protocol was developed for the anatomopathologic examination of the surgical specimens in this study. All specimens were examined grossly and photographed before formalin fixation. The quality of TME was assessed according to Nagtegaal et al. (15). The outer surface of the rectum was inked with different colors, as follows: blue, anterior wall; green, posterior wall; yellow, right lateral wall; and black, left lateral wall (**Figure 1A**). The resected specimens were opened longitudinally to spare the tumor. The location, appearance, size, and distal margin of the residual tumor were described (**Figure 1B**). After fixation in 4% formalin for at least 48 hours, the specimens were marked from the superior, inferior, and lateral margins of the residual tumor to a distance of 4 cm in each direction (**Figures 1C, D**). In cases where there was no tissue within 4 cm, the entire existing segment was analyzed. Subsequently, this area (consisting of the entire tumor or margins at the 4-cm level) was divided into 1×1 cm squares in total thickness (**Figures 1E, F**). The remainder of the surgical specimen was cut transversely and perpendicular to the longitudinal axis of the rectum up to the vascular pedicle, with an approximate thickness of 1 cm. Tissue samples were embedded in paraffin, and 3-µm-thick sections were cut and stained with hematoxylin and eosin. Microscopic examination was performed by a single pathologist in accordance with the protocol established by the College of American Pathologists (16). Tumor regression grade was analyzed using Mandard’s grading system (17). The distribution of residual disease in the rectal wall and mesorectum was examined. If residual disease was identified, the presence of RTCs in the mucosa, submucosa, muscularis propria, and subserosa/mesorectum was reported for each paraffin block analyzed from each patient. A model of RTC distribution across all layers of the rectal wall, which had been previously used in esophageal cancer (18), was adapted for use in this study as follows (**Figure 2**):

Type I – regression toward the lumen, with more tumor cells in the mucosa and submucosa.
Type II – regression toward the invasive front, with more tumor cells in the muscularis propria and subserosa/mesorectum.
Type III – concentric regression, with more tumor cells in the submucosa and muscularis propria.
Type IV – random regression, with comparable amount of tumor cells in all layers.

The pattern of tumor regression was classified as fragmented or solid and with or without microscopic intramural spread (MIS) of tumor. The fragmented pattern was defined as the presence of clusters of tumor cells separated from each other by fibrotic or normal tissue, scattered at least 1 mm apart. The solid pattern was defined as a single cluster of tumor cells surrounded by fibrotic or normal tissue (**Figure 2**). Because the solid regression pattern represents homogeneous regression with a better prognosis and pCR shares similar characteristics, the pCR cases were considered to have a solid regression pattern. MIS of tumor was defined as the presence of tumor cells underlying normal-appearing mucosa by at least 1 mm, that is, the presence of tumor cells outside the boundaries of the gross residual lesion in any direction (19). MIS was measured when present.

### 2.2 Immunohistochemistry

Tissue samples of rectal adenocarcinoma, obtained from pre-neoadjuvant biopsies, were fixed in formalin and embedded in paraffin for histological analysis. Serial 3-µm-thick histological sections were cut from each paraffin block, stained with hematoxylin and eosin, and reviewed to select the sites for removal of the tissue cylinders used to construct the tissue microarray.

#### 2.2.1 Assessment for CD8 and CD3

CD8 and CD3 staining was positive when detected in the cytoplasm or cell membrane of tumor-infiltrating lymphocytes (TILs), and the samples were examined by microscopy (NIKON Eclipse 55i) according to the intensity of CD8+ and CD3+ TILs. The samples were examined at 40× magnification, and the area with the highest density of CD8+ and CD3+ TILs adjacent to the neoplastic cells was counted at 400× magnification (number of CD8+/CD3+ TILs). The average number of CD8+/CD3+ TILs in 5 high-power fields was included in the evaluation. A count of zero CD8+/CD3+ TILs in a high-power field received a score of 0, a count of 1–3 CD8+/CD3+ TILs received a score of 1, a count of 4–10 CD8+/CD3+ TILs received a score of 2, and a count of > 10 CD8+/CD3+ TILs received a score of 3 (20). Scores of 0 and 1 were defined as low CD3 and CD8 T-cell infiltration, and scores of 2 and 3 were defined as high CD3 and CD8 T-cell infiltration.

#### 2.2.2 Assessment for PD-L1

A modified H-score (MHS) was used to assess tumor PD-L1 expression with the anti-PD-L1 antibody clone 22C3. This method sums up the expression of tumor cells and mononuclear inflammatory cells within tumor that express PD-L1 as membrane staining, with different weights for each intensity of the PD-L1 expression: weak (1+), moderate (2+), and strong (3+). The final value is calculated using the following formula: MHS = [(% positive cells at 1+)×1]+[(% positive cells at 2+)×2]+[(% positive cells at 3+)×3], with the result ranging from 0 to 300 and being considered positive when MHS ≥ 1 (21).

#### 2.2.3 Assessment for expression of DNA repair enzymes

Regarding immunoreactivity for MLH1, MSH2, MSH6, and PMS2, the result is considered negative (loss of expression) when there is no nuclear staining in tumor cells in the presence of nuclear staining in epithelial cells and lymphocytes.

### 2.3 Statistical analysis

The number of patients with rectal adenocarcinoma receiving neoadjuvant chemoradiotherapy during the study period determined the sample size. Poisson regression with robust variance was used to determine demographic and clinicopathologic factors associated with the occurrence of fragmentation and type I distribution pattern, using prevalence ratio (PR) and the respective 95% CI as the effect measure. Bivariate and multivariate analyses were performed. Initially, simple Poisson models were adjusted for each variable, and those with a *P* value < 0.05 were included in the multivariate Poisson model. The variables were then adjusted using a stepwise procedure, and only those with a *P* value < 0.05 were retained in the final model. Multicollinearity among the independent variables was assessed, and a tolerance value > 0.60 indicated the presence of multicollinearity. *P* values < 0.05 were considered statistically significant. Data were analyzed using SAS, version 9.4.

## 3 Results

### 3.1 Demographic and clinicopathologic data

Of 45 individuals eligible for the study, 5 were excluded: 4 achieved a cCR and were offered the watch-and-wait strategy, and 1 showed loss of MLH1 and PMS2 expression. Therefore, the sample consisted of 40 patients with a median age of 66 years (range, 36 to 83 years); 23 (57.5%) were men. Demographic and clinicopathologic data are shown in **Table 1**.

**Table 1:** Demographic and clinicopathologic characteristics of patients.

Variable	Frequency (n = 40)	Percentage
Sex		
Female	17	42.5
Male	23	57.5
Age		
< 60 years	17	42.5
≥ 60 years	23	57.5
Height		
≤ 5 cm	20	50.0
> 5 cm	20	50.0
CEA level		
≤ 5.0 ng/mL	26	65.0
> 5.0 ng/mL	14	35.0
Initial clinical staging		
II	6	15.0
III	34	85.0
Pathologic staging		
0 and I	19	47.5
II and III	21	52.5
CD3 and CD8 T-cell infiltration		
Low	16	37.5
High	24	55.0
PD-L1 expression		
Present	3	7.5
Absent	37	92.5

CEA, carcinoembryonic antigen.

Twenty-eight patients (70.0%) received all 6 cycles of CAPOX chemotherapy. Six patients received 5 cycles and 6 patients received 4 cycles due to toxicity (neuropathy and neutropenia). On restaging, there were 14 stage I, 17 stage II, and 9 stage III cases. Downstaging occurred in 29 patients (72.5%), and downsizing ≥ 50% occurred in 23 (57.5%).

The time interval between the start of radiotherapy and surgery ranged from 20 to 35 weeks, with a median of 28 weeks. In 32 cases, this interval was ≤ 30 weeks. Low anterior resection was performed in 32 cases, including 1 intersphincteric resection and 1 total pelvic exenteration. Abdominoperineal resection was performed in 8 cases, including 1 case of combined sacrectomy. The pathologic staging of the surgical specimens was as follows: stage I, 14 cases; stage II, 9 cases; and stage III, 12 cases. Five patients achieved a pCR. Lymphovascular invasion was diagnosed in 15 cases, and perineural invasion in 10 cases.

### 3.2 Pattern of tumor regression and microscopic intramural spread

A fragmented pattern was identified in 18 patients (45.0%), and a solid pattern in 22 (55.0%). MIS was identified in 25 patients (62.5%), extending from 1 to 18 mm (median, 4 mm). Seven cases of solid regression pattern showed MIS, ranging from 1 to 3 mm. All cases of fragmented regression pattern, except one, showed MIS. Within the fragmented pattern, 4 cases had MIS of 4–8 mm, and the remaining cases had MIS ≥ 10 mm. When the value of 1 cm, easily applicable to clinical practice, was used to dichotomize this variable, there were 14 cases (35.0%) of MIS ≥ 10 mm. All cases of MIS ≥ 10 mm were within the fragmented pattern.

After adjusting the multivariate Poisson model, the following variables were associated with the occurrence of a fragmented pattern of tumor regression: CEA levels > 5 ng/mL, downsizing < 50%, residual mucosal abnormality > 20 mm, and anatomopathologic lymph node involvement (ypN+) (*P *< 0.05) (**Table 2**).

**Table 2:** Distribution of study variables according to the Poisson regression model with robust variance for the occurrence of fragmentation (n = 40).

Variable (Frequency n = 40/percentage)	Unadjusted		Adjusted	
	PR (95% CI)	*P* value	PR (95% CI)	*P* value
Stenosis (n/%)		0.0329	–	–
No (25/62.5)	1	–	–	–
Yes (15/37.5)	2.08 (1.06–4.09)	0.0329	–	–
Passable by the colonoscope (n/%)		0.0236	–	–
No (8/20.0)	1	–	–	–
Yes (32/80.0)	2.00 (1.10–3.64)	0.0236	–	–
Extramural venous invasion – Initial staging (n/%)		< 0.0001	–	–
No (33/82.5)	1	–	–	–
Yes (7/17.5)	2.47 (1.67–3.64)	< 0.0001	–	–
Lymphovascular invasion (n/%)		0.0015	–	–
No (25/62.5)	1	–	–	–
Yes (15/37.5)	3.33 (1.59–7.00)	0.0015	–	–
Tumor deposits in the mesorectum – AP (n/%)		< 0.0001	–	–
No (34/85.0)	1	–	–	–
Yes (6/15.0)	2.83 (1.80–4.47)	< 0.0001	–	–
Microscopic intramural spread (n/%)		< 0.0001	–	–
< 10 mm (26/65.0)	1	–	–	–
≥ 10 mm (14/35.0)	6.50 (2.64–16.01)	< 0.0001	–	–
Pathologic T stage (n/%)		0.0036	–	–
T0, T1, and T2 (19/47.5)	1	–	–	–
T3 and T4 (21/52.5)	7.24 (1.91–27.44)	0.0036	–	–
Pathologic N+ stage (n/%)		0.0001		0.0017
No (28/70.0)	1	–	1	–
Yes (12/30.0)	3.67 (1.89–7.12)	0.0001	2.29 (1.36–3.85)	0.0017
Downsizing (n/%)		0.0005		0.0052
≥ 50% (23/57.5)	1	–	1	–
< 50% (17/42.5)	6.76 (2.32–19.71)	0.0005	4.03 (1.51–10.71)	0.0052
CEA level (n/%)		0.0131		0.0095
≤ 5.0 ng/mL (26/65.0)	1	–	1	–
> 5.0 ng/mL (14/35.0)	2.32 (1.19–4.51)	0.0131	2.27 (1.22–4.23)	0.0095
CD3 and CD8 T-cell infiltration (n/%)		0.0021	–	–
High (24/60.0)	1	–	–	–
Low (16/40.0)	10.27 (2.72–38.75)	0.0006	–	–
Tumor size – Restaging MRI (n/%)		< 0.0001	–	–
> 6 cm (2/5.0)	1	–	–	–
≤ 6 cm (38/95.0)	2.37 (1.64–3.45)	< 0.0001		
Size of residual mucosal abnormality (n/%)		< 0.0001		0.0484
≤ 20 mm (24/60.0)	1	–	1	–
> 20 mm (16/40.0)	2.57 (1.71–3.87)	< 0.0001	2.18 (1.01–4.73)	0.0484

PR, prevalence ratio; AP, anatomopathologic examination; CEA, carcinoembryonic antigen; MRI, magnetic resonance imaging.

### 3.3 Distribution pattern of residual tumor cells

For patients who did not achieve a pCR (n = 35), the distribution of RTCs in each layer of the rectal wall was as follows: 29 patients (82.8%) had RTCs in the mucosa; 31 (88.5%) in the submucosa; 30 (85.7%) in the muscularis propria; and 20 (57.1%) in the subserosa/perirectal fat. All 3 patients staged as ypT1 had RTCs in the mucosa (100%) and in the submucosa (100%). Among 11 patients staged as ypT2, RTCs were found in the mucosa in 10 (90.9%), in the submucosa in 9 (81.8%), and in the muscularis propria (100%). Among 20 patients staged as ypT3, RTCs were found in the mucosa in 15 (75.0%), in the submucosa in 18 (90.0%), in the muscularis propria in 19 (95.0%), and in subserosa/mesorectum (100%). The only patient staged as ypT4 (100%) had RTCs in the mucosa, submucosa, muscularis propria, and subserosa/mesorectum. According to the model of RTC distribution used in this study, 11 cases (31.5%) were classified as type I, 14 (40.0%) as type II, 10 (28.5%) as type III, and none (0%) as type IV.

After adjusting the multivariate Poisson model, the type I distribution pattern was significantly associated with having received all 6 cycles of CAPOX chemotherapy and absence of MIS of tumor (*P *< 0.05) (**Table 3**).

**Table 3:** Distribution of study variables according to the Poisson regression model with robust variance for type I distribution pattern (n = 35).

Variable (Frequency n=40/percentage)	Unadjusted		Adjusted	
	PR (95% CI)	*P* value	PR (95% CI)	*P* value
Consolidation TNT CAPOX 6 cycles (n/%)		0.0494	–	0.0053
No (12/30.0)	1	–	1	–
Yes (28/70.0)	2.41 (1.00–6.00)	0.0494	2.95 (1.38–6.32)	0.0053
Microscopic intramural spread (n/%)		0.0008		< 0.0001
Yes (25/62.5)	1	–	1	–
No (15/37.5)	6.67 (2.21–20.14)	0.0008	7.41 (2.77–19.85)	< 0.0001
Downsizing (n/%)		0.0237	–	–
< 50% (17/42.5)	1	–	–	–
≥ 50% (23/57.5)	9.44 (1.35–66.10)	0.0237	–	–
Differentiation grade (n/%)		0.0494	–	–
Moderately differentiated (31/77.5)	1	–	–	–
Well differentiated (9/22.5)	2.41 (1.00–6.00)	0.0494	–	–
MRI TRG (n/%)		0.0110	–	–
1 and 2 (8/20.0)	2.91 (1.28–6.61)	0.0110	–	–
3 and 4 (32/80.0)	1	–	–	–
Pathologic TRG (n/%)		0.0020	–	–
1 and 2 (13/32.5)	4.05 (1.67–9.83)	0.0020	–	–
3 and 4 (27/67.5)	1	–	–	–

PR, prevalence ratio; TNT, total neoadjuvant therapy; MRI, magnetic resonance imaging; TRG, tumor regression grade.

## 4 Discussion

For rectal cancer, the pattern of tumor regression is classified as solid or fragmented. The solid pattern represents homogeneous regression and consists of a single cluster of RTCs, being associated with a better prognosis. The fragmented pattern is characterized by tumor heterogeneity and consists of areas of complete response interspersed with tumor cell clusters, being associated with a worse prognosis. In the fragmented pattern, tumor cells are commonly found outside the boundaries of the gross residual lesion (MIS of tumor) (22). Since little is known about the factors that drive the development of one regression pattern over the other and that a possible explanation may lie in the relationship between tumor cells and the patient’s immune response, we performed immunostaining for CD3 and CD8 T-cell infiltration and PD-L1 expression in pre-neoadjuvant biopsy samples (23).

The importance of investigating the pattern of tumor regression and distribution of RTCs lies in the possibility of producing knowledge to be used in rectum-sparing strategies, as well as in radical surgery. Therefore, in rectal tumors with a fragmented regression pattern, due to the risk of leaving RTCs, the resection margins should be calculated based on the boundaries of the gross lesion before neoadjuvant therapy rather than on the residual lesion after chemoradiotherapy. Moreover, understanding the distribution of RTCs in the rectal wall and mesorectum may indicate the points where to increase imaging resolution, as well as the sites where to perform the biopsy after neoadjuvant therapy.

Our study identified a fragmented tumor regression pattern in 45% of cases. The variables associated with this pattern were residual mucosal abnormality > 20 mm, CEA levels > 5 ng/mL, downsizing < 50%, and anatomopathologic lymph node involvement. The size of residual mucosal abnormalities has been associated with pathologic T stage (ypT), with increasing diameters being associated with more advanced ypT (24). A similar relationship exists between the occurrence of a fragmented regression pattern and ypT, and therefore it seems reasonable to associate the size of residual mucosal abnormalities with fragmentation. The occurrence of downsizing < 50% reflects a poorer response to neoadjuvant therapy, which is also characteristic of fragmented regression (8, 9). Above-normal CEA levels have been associated with an increased likelihood of metastatic disease, increased tumor volume, and poor prognosis, characteristics also common to the fragmented pattern (25). The relationship between fragmented regression and anatomopathologic lymph node involvement has been previously described, where the occurrence of a fragmented pattern is associated with decreased lymph node sterilization in the mesorectum (8). Therefore, all 4 variables associated with the fragmented pattern in the current study indicate less tumor regression in response to the action of neoadjuvant chemoradiotherapy and poorer prognosis, factors consistent with this regression pattern. An interesting aspect is that CEA levels, as well as downsizing and the size of residual mucosal abnormalities, can be easily measured and used for preoperative prediction of the regression pattern.

MIS of tumor occurred in most patients (n = 25, 62.5%), extending from 1 to 18 mm. As expected, all but one case of fragmented regression showed MIS. Furthermore, the fact that all cases of MIS ≥ 10 mm showed a fragmented regression pattern leads us to believe that these cases represent situations of exuberant fragmented regression. However, we identified that nearly one-third of patients with solid regression (7 out of 22) had MIS, which was unexpected by definition. It is worth mentioning that these measurements were obtained from formaldehyde-fixed specimens, which are known to have no perfect correlation with *in vivo* tissue. Based on these data, we believe that a margin of 20 mm distally and laterally is sufficient for radical surgery.

A retrospective analysis of local excision specimens, from a sample of patients with residual tumors up to 3 cm in diameter, identified a fragmented regression pattern in 37% and the presence of MIS in 53% of the specimens, with a maximum spread of 7.2 mm (19). These rates are slightly lower than ours, but there are differences between the two studies. In the current study, cases were not excluded according to the size of residual tumor and the neoadjuvant chemoradiotherapy regimen was different. Another retrospective analysis of patients undergoing TME identified higher rates of fragmented pattern (80% of cases) and MIS (71% of surgical specimens) (26) than those reported in the current study. However, the largest extension of MIS was measured in our study (9 × 18 mm). These differences may be explained by the different study designs (prospective vs retrospective), where, in our study, we used a dedicated anatomopathologic examination protocol for a complete sampling of the tumor and surrounding area, controlling for direction and thickness. No less important, the chemoradiotherapy regimens and timing of surgery were different. In a meta-analysis consisting of 5 studies, with 349 patients in total, MIS was identified in 20% of patients, with an extension of 0 to 20 mm (27). Our discrepant results may be explained by differences in neoadjuvant chemoradiotherapy regimens, timing of surgery, and surgical techniques used.

It is our understanding that the investigation of how a tumor regresses should be accompanied by an investigation of how it is distributed, because altogether this information can impact the management of rectal cancer. As in previous studies, in our study the presence of RTCs was described across the different layers of the rectal wall. Duldulao et al. (11), in a secondary analysis of data obtained from a prospective phase II study of patients with stage II or stage III rectal cancer, found RTCs in the mucosa in 14% of cases, in the submucosa in 42%, and in the muscularis propria in 60%. Our findings are different, with rates of 82%, 88%, and 85%, respectively. Possible explanations for such a difference are suggested. Our study was designed and conducted with the aim of investigating the pattern of regression and distribution of RTCs using a detailed and comprehensive anatomopathologic examination protocol. There is no information in this regard in the study by Duldulao et al. Perhaps the discrepancy between anatomopathologic examination methods can explain the difference. Other factors may help explain this disparity, such as the use of different neoadjuvant chemoradiotherapy regimens and different timing of surgery.

It is important to note that the identification of RTCs in the mucosa, or in any other layer of the rectal wall, does not mean that they will be found in this layer in all histological sections of the tumor. Contrariwise, there were cases of RTCs in the most superficial layers in only one or two histological sections, which did not occur in dozens of other sections. In these same cases, a large number of RTCs were commonly identified in the deeper layers. Therefore, it is our understanding that an endoscopic biopsy, with a sampling of only the mucosa, will be unlikely to identify RTCs in more than 80% of cases.

Simply describing the presence of RTCs in a layer does not indicate where these cells will be most concentrated. It would therefore be interesting to use a classification that represents the distribution pattern of RTCs. To date, to our knowledge, there is no such classification for rectal cancer. In the current study, we used a classification that had been previously applied to esophageal cancer (18). It consists of 4 distribution patterns: type I (luminal), with RTCs concentrated in the mucosa and submucosa; type II (invasive front), with RTCs concentrated in the muscularis propria and subserosa/mesorectum; type III (concentric), with RTCs concentrated in the submucosa and muscularis propria; and type IV (random), with comparable amount of RTCs in all layers.

In this study, we found similar rates of type I (31.5%), type II (40.0%), and type III (28.5%) distribution, with no type IV cases. In multivariate analysis, having received all 6 cycles of CAPOX chemotherapy and absence of MIS were associated with type I distribution. Assuming that the type I distribution pattern represents situations of increased tumor response and sensitivity to neoadjuvant therapy, it seems reasonable to consider its association with the completion of all cycles of consolidation chemotherapy, where more drugs have been administered and the interval between the start of radiotherapy and surgery is longer. The association between type I distribution pattern and absence of MIS is also possible, as both represent situations of favorable tumor response to neoadjuvant therapy. Given that the type I distribution pattern represents tumor regression toward the lumen (more superficial layers) and is associated with the absence of MIS (gross and microscopic responses are similar), the occurrence of this distribution pattern fulfills some of the criteria for local excision. Unfortunately, we were unable to compare our results with those of previous studies on the distribution pattern of RTCs in rectal cancer because there is none.

We acknowledge that this study has limitations. The study was conducted in a real-world setting, making it difficult to accurately control for some variables, such as timing of restaging, completion of all cycles of consolidation chemotherapy, and timing of surgery. However, strengths of this study include its prospective design with the use of an exclusive and comprehensive anatomopathologic examination protocol. In addition, the anatomopathologic examination was performed by a pathologist dedicated to gastrointestinal tract pathology, which reinforces the reliability of the results. The study also describes the pattern of regression and distribution of RTCs in a context of short-course radiotherapy followed by consolidation chemotherapy, along the lines of the RAPIDO trial (14); to date, there are no similar data in the literature.

## 5 Conclusion

After the use of short-course radiotherapy followed by consolidation chemotherapy, we could identify a fragmented regression pattern in almost half of the cases. We also identified factors associated with the occurrence of this pattern of tumor regression. Regarding the distribution of RTCs across the different layers of the rectal wall, we could identify RTCs in the most superficial layers in most cases. However, their concentration (distribution pattern) had a homogeneous distribution.

## Data availability statement

The original contributions presented in the study are included in the article/supplementary material. Further inquiries can be directed to the corresponding author.

## Ethics statement

The studies involving humans were approved by Research Ethics Committee of the Institute of Strategic Health Care Management of the Federal District – Hospital de Base of the Federal District, Brazil. The studies were conducted in accordance with the local legislation and institutional requirements. The participants provided their written informed consent to participate in this study.

## Author contributions

AG: Conceptualization, Data curation, Formal analysis, Investigation, Methodology, Validation, Visualization, Writing – original draft, Writing – review & editing. DB: Conceptualization, Data curation, Formal analysis, Investigation, Validation, Visualization, Writing – original draft, Writing – review & editing. MC: Data curation, Formal analysis, Validation, Writing – review & editing. ST: Data curation, Formal analysis, Validation, Writing – review & editing. OF: Data curation, Formal analysis, Validation, Writing – review & editing. FL: Supervision, Writing – review & editing. DG: Supervision, Writing – review & editing. JS: Conceptualization, Data curation, Formal analysis, Writing – original draft, Writing – review & editing.
